# The Role of Peroxisome Proliferator-Activated Receptors in Endometrial Cancer

**DOI:** 10.3390/ijms24119190

**Published:** 2023-05-24

**Authors:** Iason Psilopatis, Kleio Vrettou, Constantinos Troungos, Stamatios Theocharis

**Affiliations:** 1 First Department of Pathology, Medical School, National and Kapodistrian University of Athens, 75 Mikras Asias Street, Bld 10, Goudi, 11527 Athens, Greecekliovr1@gmail.com (K.V.); 2 Department of Biological Chemistry, Medical School, National and Kapodistrian University of Athens, 75 Mikras Asias Street, Bld 16, Goudi, 11527 Athens, Greece

**Keywords:** peroxisome proliferator-activated receptor, PPAR, endometrial cancer

## Abstract

Endometrial carcinoma is the most common malignant tumor of the female genital tract in the United States. Peroxisome proliferator-activated receptors (PPARs) are nuclear receptor proteins which regulate gene expression. In order to investigate the role of PPARs in endometrial cancer, we conducted a literature review using the MEDLINE and LIVIVO databases and were able to identify 27 relevant studies published between 2000 and 2023. The PPARα and PPARβ/δ isoforms seemed to be upregulated, whereas PPARγ levels were reported to be significantly lower in endometrial cancer cells. Interestingly, PPAR agonists were found to represent potent anti-cancer therapeutic alternatives. In conclusion, PPARs seem to play a significant role in endometrial cancer.

## 1. Introduction

Endometrial cancer represents the most common malignant female genital tract tumor in the United States. According to the American Cancer Society, about 66,200 new cases of cancer of the uterine body will be diagnosed and about 13,030 women will die from cancers of the body of the uterus in the United States in 2023 [1]. Endometrial cancer most frequently affects postmenopausal women aged 55–64, with the median age at diagnosis being 63 years [2]. Endometrial cancer may be subdivided into two distinct histopathologic categories: type I endometrial carcinoma originating from atypical hyperplasia of the endometrium and type II endometrial cancer of non-endometrioid histology [3]. Type I endometrial carcinoma is directly associated with long-term exposure to high estrogen levels and correlates with Phosphatase and TENsin homolog (PTEN) inactivation by mutation, microsatellite instability, as well as mutations of Kirsten RAt Sarcoma virus (KRAS), β-catenin, and/or human MutL Homolog 1 (hMLH1)/MutS Homolog 2 (MSH2). Type II endometrial cancer is mostly estrogen independent, arises from atrophic endometrium in postmenopausal patients, and is associated with p53 mutations, display inactivation of p16 and E-cadherin, alongside Human Epidermal growth factor Receptor 2 (HER2) amplification [4,5]. While surgery is suggested as a monotherapy for low-risk endometrial malignancies, adjuvant chemotherapy needs to be offered to patients with high–intermediate- or high-risk endometrial cancer, as well as advanced and/or recurrent disease [6]. Combined chemotherapy with carboplatin and paclitaxel embodies the first-line chemotherapeutic regimen, followed by chemotherapeutic agents including doxorubicin, cyclophosphamide, or cisplatin [7].

In spite of the reported high response rates to chemotherapy, the duration of response only ranges between four and eight months [8,9], whereas the 5-year overall survival amounts to 84%, according to the American Cancer Society [10]. Nevertheless, the prognosis for women with advanced disease remains grim, with 5-year survival rates dropping to 20%, in cases of distant metastasis [10]. Such statistics render a better understanding of the endometrial cancer pathophysiology, as well as the identification of alternative treatment targets for its effective therapy, imperative.

Peroxisome proliferator-activated receptors (PPARs) are fatty acid-activated nuclear receptors [11]. The three PPAR isoforms PPARα, PPARβ/δ, and PPARγ, exhibit discrete metabolic regulatory activities, tissue distribution, and ligand-binding properties [12,13]. In their capacity to form heterodimers with the retinoid X receptors (RXRs) and bind to specific DNA response elements within promoters, PPARs represent ligand-regulated transcription factors that competently induce or repress the expression of their target genes [14]. With the size of the PPAR ligand binding cavity being significantly larger than that of other nuclear receptors, numerous natural and synthetic ligands may attach and trigger an exchange of co-repressors for co-activators, thereby stimulating the functions of PPARs [15]. Fatty acid disposition and metabolism, diverse cellular biology functions, cell differentiation, energy homeostasis, as well as immunity mechanisms, are only a few of the functions that PPARs regulate [16,17]. More precisely, PPARα is highly expressed in the liver, intestine, heart, kidneys, skeletal muscles, and brown adipose tissue, and co-determines fatty acid metabolism. PPARβ/δ shows ubiquitous expression and controls fatty acid oxidation, alongside the regulation of blood glucose and cholesterol levels. PPARγ displays the highest expression in adipose cells and significantly contributes to lipid biosynthesis, lipoprotein metabolism, adipogenesis, as well as insulin sensitivity [15,18].

**Figure 1:**
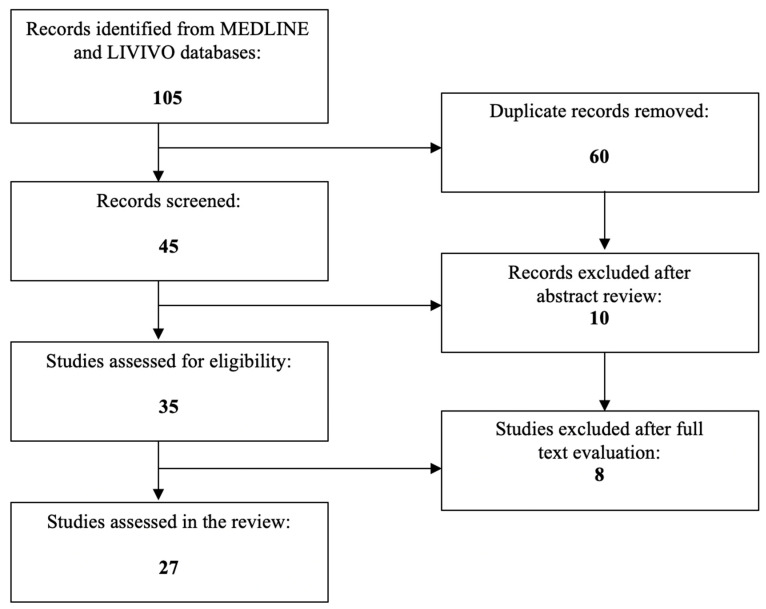
PRISMA flow diagram visually summarizing the screening process.

To date, PPARs have been described as playing an important role in numerous cancer entities and gynecological health conditions [19,20,21,22]. The present literature review was conducted with a view of specifically investigating the role of PPARs in endometrial cancer. The literature search was conducted using the MEDLINE and LIVIVO databases. Solely original research articles and scientific abstracts written in the English language, which explicitly reported on the role of PPARs in endometrial cancer, were included in the data analysis. Studies focusing on the involvement of PPARs in (atypical) endometrial hyperplasia or uterine sarcoma were excluded. The search terms “peroxisome proliferator-activated receptor”, “PPAR”, and “endometrial cancer” were employed, and we were able to identify a total of 45 articles published between 1996 and 2023, after the exclusion of duplicates. A total of 10 works were discarded in the initial selection process after abstract review. The full texts of the remaining 35 publications were evaluated, and after detailed analysis, a total of 27 relevant studies published between 2000 and 2023, that met the inclusion criteria, were selected for the literature review. Figure 1 presents an overview of the aforementioned selection process.

## 2. The Role of PPARs in Endometrial Cancer

Four study groups have, to date, examined the role of different PPAR isoforms in endometrial cancer.

Modesitt et al. analyzed the endometrial and adipose tissue from endometrial cancer patients and reported PPAR upregulation in both endometrial and visceral adipose tissue, but downregulation in subcutaneous adipose tissue [23]. Furthermore, Tang et al. used The Cancer Genome Atlas (TCGA) and identified numerous somatic changes in *PPAR*-related genes in women suffering from endometrial cancer. Notably, copy number variations and nucleotide mutations in the exon regions differentially influenced disease prognosis and overall survival, with *Diazepam Binding Inhibitor* (*DBI*), *Carnitine Palmitoyl Transferase 1A* (*CPT1A*), *CYP27A1*, and *Malic Enzyme 1* (*ME1*), incorporating the four most significant *PPAR*-related genes linked to endometrial cancer patient prognosis [24]. Moreover, Knapp et al. gathered 35 endometrial carcinoma tissue samples and proposed a significant upregulation of PPARα and PPARβ/δ, but a significant downregulation of PPARγ protein expression in endometrial cancer cells. Notably, PPAR immunohistochemical expression was mainly restricted to the (peri-)nuclear cellular regions [25]. Nickkho-Amiry et al. first explored the expression patterns of the three PPAR isoforms in endometrial cancer tissue samples and suggested a significant upregulation of PPARα and PPARβ/δ, but a significant downregulation of PPARγ. In the Ishikawa and HEC-1A endometrial cancer cell lines, both the PPARα ligand fenofibrate and the PPARγ agonist ciglitazone impressively induced apoptosis and inhibited cellular proliferation. However, only fenofibrate reduced Vascular Endothelial Growth Factor (VEGF) concentration in vitro [26].

## 3. The Role of PPARα in Endometrial Cancer

So far, two studies have focused on the role of PPARα in endometrial cancer. 

Holland et al. used complementary DNA (cDNA) microarrays in order to explore the transcriptome of endometrial carcinoma tissues and suggested elevated PPARα and PPARγ levels in endometrial cancer. In addition, the treatment of cultured endometrial carcinoma cells with the PPARα activator fenofibrate resulted in significantly downregulated cancer cell proliferation, alongside profound apoptosis induction [27]. Similarly, Saidi et al. applied fenofibrate to Ishikawa endometrial cancer cells, which, in doses above 25 μM and in synergy with retinoic acid, efficiently suppressed cellular proliferation and promoted apoptotic cell death. Interestingly enough, fenofibrate did not, nevertheless, manage to consistently inhibit endometrial carcinoma growth in a treated nude mouse model [28].

## 4. The Role of PPARβ/δ in Endometrial Cancer

Tong et al. measured high PPARβ/δ expression levels in eleven endometrial adenocarcinoma tissue samples [29], while Ma et al. investigated the role of PPARβ/δ in endometrial cancer and observed that this PPAR isoform exerted significant growth inhibitory and apoptotic effects in the endometrial cancer cell lines Ishikawa, RL95-2, and Sawano. Interestingly enough, high PPARβ/δ levels correlated with increased Protein Kinase B and Glycogen Synthase Kinase (GSK) 3β dephosphorylation, alongside enhanced β-catenin phosphorylation [30].

## 5. The Role of PPARγ in Endometrial Cancer

PPARγ is undoubtedly the most studied PPAR isoform in endometrial cancer.

Gu et al. reported that the human endometrial carcinoma cell lines Ishikawa and RL95-2 had increased or decreased PPARγ expression levels, following overexpression of ubiquitin C or specificity protein 1, respectively. Notably, the application of melatonin to Melatonin Receptor 1B (MTNR1B)-overexpressing Ishikawa and RL95-2 cells also led to higher PPARγ levels [31]. 

In the context of *PPARγ* gene polymorphisms, Paynter et al. genotyped the *PPARγ* Pro12Ala polymorphism in a cohort of invasive endometrial cancer cases and concluded that the aforementioned polymorphism did not seem to mediate circulating estrogen levels or endometrial cancer susceptibility [32]. Nonetheless, Smith et al. found the *PPARγ* H449H variant to be overrepresented in endometrial carcinoma patients [33].

Huang et al. collected tissue samples from women suffering from endometrial cancer and underlined a significant negative association between PPARγ and estrogen-related receptor-α expression. More accurately, both PPARγ and estrogen-related receptor-α regulated numerous protein-coding genes responsible for the apoptosis pathway, were reciprocally inhibited in endometrial cancer cells, and competed to promote or prevent cell proliferation and death through the B-cell lymphoma 2 (Bcl2)/Caspase3 pathway. Most importantly, a PPARγ/estrogen-related receptor-α ratio ≤1.86 represented an independent risk factor for endometrial carcinogenesis, whereas women with PPARγ(−)/estrogen-related receptor-α(+) were found to exhibit the worst overall survival and disease-free survival rates [34]. Zhang et al. underlined that PPARγ expression levels significantly correlated with estrogen receptor α expression levels, alongside endometrial cancer pathological grade and clinical stage. Additionally, transfection of KLE and ECC-1 cells with PPARγ short-interfering RNA suppressed their migratory, invasive, and proliferative, capacities [35].

Focusing on the PPARγ Coactivator 1 alpha (PGC-1α), Cormio et al. employed 52 patient-derived endometrial tissue samples and highlighted the direct association of the enhanced mitochondrial biogenesis in type I endometrial carcinoma with the increased PGC-1α signaling pathway [36]. Accordingly, Ren et al. collected 40 cases of endometrial carcinoma and reported a significantly more profound PGC-1α mRNA expression in high-grade malignant tissues, especially those of endometrial cancer patients with type 2 diabetes. In addition, the PGC-1α mRNA levels showed a positive association with the concentrations of the estrogen-related receptor gamma, the pyruvate kinase, and the isocitrate dehydrogenase [37]. On the contrary, Wersäll et al. described that PGC-1α expression was significantly downregulated in endometrial cancer tumor samples, but did not correlate with patient survival, grade, stage, p53 status, Ki-67, or clinical resistance [38]. In 2016, Yang et al. published two original research articles on the role of PGC-1α in endometrial cancer. More precisely, PGC-1α synergized via the mitochondrial pathway with estrogen to ensure endometrial cancer cell survival [39], whereas PGC-1α knockdown promoted apoptotic cell death through a reduction in Bcl2 and an increase in Bcl2-associated X (Bax) expression [40]. Yoriki et al. concluded that estrogen-related receptor-α/PGC-1α overexpression in Ishikawa and HEC-1A endometrial cancer cells decreased E-cadherin, but upregulated the expression of vimentin, Snail, and Zinc finger E-box Binding homeobox 1 (ZEB1) after exposure to Transforming Growth Factor beta (TGF-β), enhancing cancer cell motility via cancer–stromal interactions [41].

Importantly, a great number of researchers examined the therapeutic effects of diverse PPARγ ligands in endometrial cancer treatment. Ota et al. highlighted that PPARγ immunoreactivity was significantly lower in endometrial carcinoma, positively correlated with the p21 expression, but negatively correlated with the patients’ body mass index. Notably, the PPARγ agonist 15-Deoxy-Δ^12,14^-prostaglandin J2 successfully inhibited cancer cell proliferation and promoted p21 mRNA expression in the Ishikawa, Sawano, and RL95-2 endometrial carcinoma cell lines [42]. Similarly, Li et al. treated the endometrial cancer cell lines HHUA, Ishikawa, and HEC-59, with the PPARγ ligand 15-Deoxy-Δ^12,14^-prostaglandin J2 and reported significant growth inhibitory and apoptotic effects in all three studied cancer cell lines. As far as gene expression changes are concerned, the Aldo-Keto Reductase family 1 member C3 (AKR1C3) was found to be upregulated, whereas both the Anterior Gradient Homolog 3 (AGR3) and the Nitric Oxide Synthase 2A (NOS2A) were seemingly downregulated in all three endometrial cancer cell lines [43]. Hong et al. examined the effects of the PPARγ agonists 1,1-Bis(3′-indolyl)-1-(*p*-substituted phenyl)methanes, and its *p*-*t*-butyl (DIM-C-pPhtBu) and phenyl (DIM-C-pPhC6H5) substituents, in endometrial cancer and revealed that the PPARγ-active C-DIMs not only diminished the proliferation of HEC1A endometrial cancer cells, but also promoted the induction of apoptotic cancer cell death by reducing the mitochondrial membrane potential and inducing the release of cytochrome c, the activation of caspases, as well as the nuclear uptake of endonuclease G [44]. Furthermore, Koyama et al. treated the endometrial cancer cell lines HHUA, Ishikawa, and HEC-59, with the PPARγ ligand telmisartan, which exerted potent growth inhibitory and apoptotic effects in all three studied cancer cell lines. In HHUA cells, telmisartan application specifically resulted in the induction of DNA double-strand breaks. In vivo, telmisartan had a significant impact on human endometrial cancer growth, with negligible side effects [45]. Kumari et al. applied the PPARγ agonist pioglitazone to female Swiss albino mice with endometrial cancer and noted a significant increase in weekly body weight, improvement in mean survival time, and partial normalization of uterine tissue weight, in comparison with the standard chemotherapeutic agent, paclitaxel [46]. Accordingly, Wu et al. treated HEC-1A and Ishikawa cells with rosiglitazone, which inhibited cancer cellular growth and induced apoptosis in a dose-dependent manner [47]. Surazynski et al. treated Ishikawa cells with the PPARγ agonists troglitazone and clofibrat, which inhibited collagen biosynthesis after estrogen receptor stimulation, endorsed Nuclear Factor kappa B (NF-κB) expression, and downregulated p38 expression [48]. Last, but not least, Ruan et al. treated endometrial cancer cells with the phytoestrogen kaempferol, which induced PPARγ expression in HEC-1A and KLE cells, but inhibited PPARγ expression and PGC-1α activation in AN3 CA cells [49].

Table 1 summarizes the role of PPARs in endometrial cancer.

**Figure 2:**
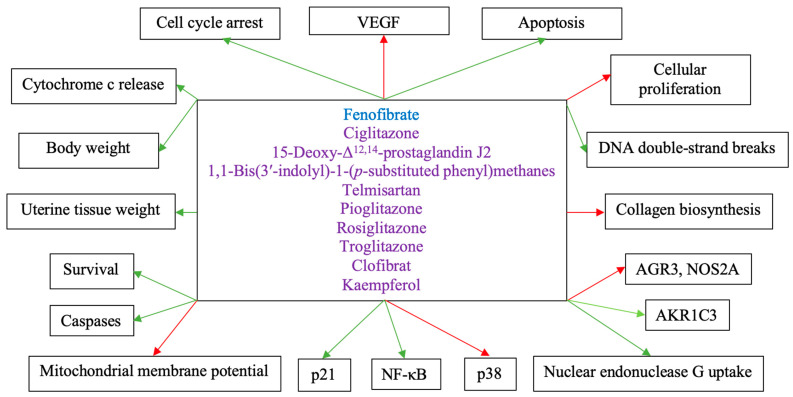
The effects of PPAR-modulating treatment agents in endometrial cancer. Blue caption: PPARα agonist. Purple caption: PPARγ agonist. Red arrows: Inhibitory effects. Green arrows: Activating effects.

Figure 2 depicts the effects of PPAR-modulating treatment agents in endometrial cancer.

## 6. The Role of PPARs in Other Uterine Cancer Entities

In addition to endometrial cancer, cervical cancer also represents another important cancer entity of the uterus. Cervical cancer originates from the cells that line the cervix, where squamous cell carcinomas develop from cells in the exocervix and adenocarcinomas arise from mucus-producing endocervical gland cells [50]. The majority of cervical cancer cases are squamous cell carcinomas that begin in the transformation zone, where the exocervix meets the endocervix [50]. In 2023, the American Cancer Society predicts approximately 13,960 new cases of invasive cervical cancer and 4310 associated deaths in the United States [51]. However, due to the widespread use of screening technology such as the Papanicolaou (PAP) and HPV tests, along with HPV vaccinations, cervical cancer is no longer a leading cause of cancer death in women [51]. Nonetheless, pre-cancerous conditions, such as dysplasia, SIL, adenocarcinoma in situ, and CIN, are still frequently diagnosed [50]. The primary risk factor for cervical (pre-)cancer is infection with high-risk types of HPV, followed by chlamydia infection, immune deficiency, smoking, chronic use of oral contraceptives, multiple full-term pregnancies (especially at a young age), a diet low in fruits and vegetables, low socioeconomic status, diethylstilbestrol use, or use of an intrauterine device [52]. Women with early stage cervical cancer or pre-cancerous conditions often do not exhibit any symptoms, while larger tumors may cause abnormal vaginal bleeding, pelvic pain, dyspareunia, or unusual vaginal discharge [53]. Treatment options for cervical cancer vary depending on the stage of the cancer and may include surgical procedures such as conization, radical trachelectomy, or hysterectomy, or radiation therapies such as external beam radiation therapy, with or without chemotherapy, depending on the patient’s desire to preserve fertility [54]. Standard treatment for locally advanced cervical cancer involves platinum-based chemoradiation, while primary chemotherapy (+/− immunotherapy) is the preferred treatment for primary metastatic disease [54].

Cervical cancer is the fourth most common cancer in women worldwide, with a 92% 5-year relative survival rate for localized cervical carcinomas [55]. However, the 5-year survival rates for patients with advanced cervical cancer are discouraging, with only 17% of patients diagnosed with invasive cervical cancer in a distant SEER stage [56]. As a result, the imperative improvement of patient’s overall survival, disease-free survival, and progression-free survival undeniably requires a better understanding of the exact pathophysiology, alongside prompt creation of new effective anti-cancer medications.

In this context, numerous study groups have investigated the role of PPARs in cervical cancer. Over the past six years, a total of six original research articles have been published on the role of PPARs in cervical cancer. In 2017, Chang et al. treated the human cervical cancer HeLa cells with the PPARγ agonists ciglitazone and troglitazone, which, in higher doses, activated caspase-3 and PARP cleavage, thereby inducing apoptotic cell death in a time- and dose-dependent way [57]. The same year, Wuertz et al. targeted the CaSki, SiHa, and HeLa cervical cancer cells with the three PPARγ agonists pioglitazone, rosiglitazone, and ciglitazone. All three agents diminished the cervical cancer cellular proliferation in a dose-dependent manner, stimulated Oil Red O accumulation, and upregulated the lipid differentiation marker adipsin. In vivo, pioglitazone even exhibited greater tumor growth inhibitory effects in comparison with standard cervical chemotherapy [58]. Similarly, Plissonnier et al. also administered the three PPARγ agonists pioglitazone, rosiglitazone, and ciglitazone to CaSki, C-33A, and HeLa cervical cancer cells. Interestingly, solely ciglitazone restored the TNF-Related Apoptosis-Inducing Ligand (TRAIL) sensitivity and prevented the E6 blocking action to induce apoptotic cervical cancer cell death both in vitro and in vivo [59]. In 2018, Tian et al. identified and validated the PPAR signaling pathway as an important biomarker related to lymph node metastasis in women with cervical cancer [60]. A year later, Hernández-Reséndiz et al. concluded that, under normoxic conditions, a decreased growth rate of mutant p53^R248Q^ overexpressing HeLa cervical cancer cells was associated with lower levels of PGC-1α [61]. 

Except for endometrial and cervical cancer, PPARs have been described to play a significant role in uterine leiomyosarcoma, as well. More precisely, Lützen et al. reported that pioglitazone prompted cell growth arrest and induced mitochondrial apoptotic cancer cell death in human uterine leiomyosarcoma cells through a PPARγ-independent pathway [62]. Furthermore, the same study group underlined that the activation of the cell membrane angiotensin receptor 2 provoked human leiomyosarcoma cell differentiation and apoptosis in a PPARγ-dependent manner [63]. Last but not least, Mineda et al. highlighted that resveratrol inhibited the proliferation and promoted the apoptosis of uterine sarcoma cells via inhibition of the Wnt signaling pathway [64].

Taken altogether, PPARs seem to play an important role not only in endometrial cancer but also in other uterine cancer entities, including cervical cancer and uterine leiomyosarcoma.

## 7. Discussion

All PPAR isoforms have been demonstrated to be expressed in the uteri of diverse mammal species [65]. The discrepancy in their expression patterns, however, might be attributed to the physiological status of each individual or species [65]. Given their differential expression during various reproductive conditions, PPARs seem to be involved in the regulation of multiple uterine secretory functions crucial for the lysis of the corpus luteum during the estrous cycle or the implantation of the embryo during pregnancy [66,67,68]. Their effects at the endometrial level may be mediated through their interactions with prostaglandins, steroids, as well as cytokines [65]. Moreover, changes in the PPAR expression profile seem to cause hormonal disturbances and to influence signal transduction during inflammatory processes occurring in the endometrium during cystic endometrial hyperplasia and pyometra [69]. Interestingly enough, the actions of PPAR agonists have been also proven to correlate with estradiol-induced proliferation and hyperplasia formation in the mouse uterus [70]. To date, no review article has, to our knowledge, been published on the role of PPARs in endometrial malignancies, and more precisely, endometrial cancer. The present work represents the most comprehensive, up-to-date review of the literature on the effects of the different PPAR isoforms in endometrial cancer genesis and progression, as well as on their capacities as potential anti-cancer treatment targets.

PPARα has been found to be upregulated in endometrial cancer cells, while the PPARα ligand fenofibrate seems to inhibit cellular proliferation, induce apoptosis and negatively influence angiogenesis in endometrial cancer. These contradictory observations may be partly explained by the fact that different endometrial cancer cell lines show differential expression of steroid hormone receptors, which can regulate cellular PPARα levels and maintain regular PPARα binding [26]. Similarly, PPARβ/δ seems to be overexpressed in endometrial cancer cells. PPARγ is undoubtedly the most studied PPAR isoform in endometrial cancer. From the studied *PPARγ* gene polymorphisms, the H449H variant seems to be overrepresented in women suffering from endometrial carcinoma. Most research groups reported PPARγ downregulation, but PGC-1α upregulation, in endometrial cancer. Concerning the therapeutic effects of PPARγ ligands, all studied agents exerted potent both anti-growth and pro-apoptotic effects in vitro and/or in vivo. Most importantly, copy number variations and nucleotide mutations in the exon regions of *PPARs* differentially influenced endometrial cancer prognosis and overall survival, while a PPARγ/estrogen-related receptor-α ratio ≤1.86 constituted an independent risk factor for endometrial carcinogenesis. Additionally, PPARγ expression levels were significantly associated with estrogen receptor α expression levels, alongside endometrial cancer pathological grade and clinical stage.

Unfortunately, no identified study has, thus far, incorporated a relatively large number of tissue samples that would have allowed for safer and unbiased results. In addition, no PPAR agonist was administered to endometrial cancer patients in the context of randomized controlled trials, which would have helped evaluate the clinical applicability/efficacy of these agents and recognize eventual unidentified toxic effects. Therefore, future studies ought to address the aforementioned limitations and, ideally, investigate the role of PPARs in type I and II endometrial cancers separately.

One limitation of the current review is the nonsystematic methodology in terms of study selection. Even though systematic literature reviews offer the most accurate strategy for the identification of relevant research works, respecting rigorous rules and standards, this approach requires a narrow research question that does not cover broader subjects, such as the role of PPARs in endometrial cancer. Another limitation is the eventual evidence selection bias, arising from publication bias, as data from statistically insignificant studies are less likely to reach publication. 

## 8. Conclusions

In summary, the present review of the literature highlights the crucial role of PPARs in endometrial cancer development and progression and underlines the therapeutic potential of PPAR agonists against endometrial cancer cells. Further systematic research is still required in this field to achieve safe and reproducible results and to comprehensively define the role of PPARs in endometrial cancer pathogenesis and therapy.

## Figures and Tables

**Table 1:** The role of PPARs in endometrial cancer.

Study	PPAR Isoform	Effect in Endometrial Cancer
Modesitt et al. [23]	Not specified	Upregulation in both endometrial and visceral adipose tissue Downregulation in subcutaneous adipose tissue
Tang et al. [24]	Not specified	Copy number variations and nucleotide mutations in the exon regions differentially influence disease prognosis and overall survival *DBI*, *CPT1A*, *CYP27A1*, and *ME1*, constitute the most significant *PPAR*-related genes linked to patient prognosis
Knapp et al. [25]	PPARα PPARβ/δ PPARγ	Upregulation of PPARα and PPARβ/δ Downregulation of PPARγ
Nickkho-Amiry et al. [26]	PPARα PPARβ/δ PPARγ	Upregulation of PPARα and PPARβ/δ Downregulation of PPARγ
Holland et al. [27]	PPARα PPARγ	Elevated PPARα and PPARγ levels
Tong et al. [29]	PPARβ/δ	High PPARβ/δ expression levels
Ma et al. [30]	PPARβ/δ	Significant growth inhibitory and apoptotic effects Increased Protein Kinase B and GSK3β dephosphorylation Enhanced β-catenin phosphorylation
Paynter et al. [32]	PPARγ	Pro12Ala polymorphism does not mediate circulating estrogen levels or endometrial cancer susceptibility
Smith et al. [33]	PPARγ	Overrepresentation of the H449H variant
Huang et al. [34]	PPARγ	Negative association between PPARγ and estrogen-related receptor-α expression Cancer cell proliferation and apoptosis regulation PPARγ/estrogen-related receptor-α ratio ≤ 1.86 represents an independent risk factor for endometrial carcinogenesis PPARγ(−)/estrogen-related receptor-α(+) status correlates with the worst overall survival and disease-free survival rates
Zhang et al. [35]	PPARγ	Significant correlation with estrogen receptor α expression levels, pathological grade and clinical stage
Cormio et al. [36]	PPARγ (PGC-1α)	Direct association with the enhanced mitochondrial biogenesis in type I endometrial carcinoma
Ren et al. [37]	PPARγ (PGC-1α)	More profound mRNA expression in high-grade malignant tissues, especially those of endometrial cancer patients with type 2 diabetes Positive association with the concentrations of the estrogen-related receptor gamma, the pyruvate kinase, and the isocitrate dehydrogenase
Wersäll et al. [38]	PPARγ (PGC-1α)	Significant downregulation No correlation with patient survival, grade, stage, p53 status, Ki-67, or clinical resistance
Yang et al. [39,40]	PPARγ (PGC-1α)	Synergy via the mitochondrial pathway with estrogen to ensure endometrial cancer cell survival
Yoriki et al. [41]	PPARγ (PGC-1α)	Decreased E-cadherin expression Upregulated vimentin, Snail, and ZEB1 expression after TGF-β exposure
Ota et al. [42]	PPARγ	Significantly lower expression Positive correlation with the p21 expression Negative correlation with the patients’ body mass index

## Data Availability

Not applicable.
